# Paired single cell analysis reveals chemotherapy resistance in osteosarcoma

**DOI:** 10.20517/cdr.2026.09

**Published:** 2026-04-21

**Authors:** Li Hu, Yaxin Zhang, Boyang Wang, Qian Liu, Feiyang Qi, Huimin Liu, Qinghua Li, Zhiqing Zhao, Haijie Liang, Xingyu Liu, Zhiye Du, Jichuan Wang

**Affiliations:** ^1^Familial & Hereditary Cancer Center, Peking University Cancer Hospital & Institute, Key Laboratory of Carcinogenesis and Translational Research (Ministry of Education), Beijing 100142, China.; ^2^Musculoskeletal Tumor Center, Beijing Key Laboratory for Musculoskeletal Tumors, Peking University People’s Hospital, Beijing 100044, China.; ^3^Multidisciplinary Diagnosis and Treatment Center for Bone Tumors, Peking University Shougang Hospital, Beijing 100144, China.; ^4^Department of Biochemistry and Molecular Biology, School of Basic Medical Sciences, Peking University International Cancer Institute, Peking University Health Science Center, Beijing 100191, China.; ^5^Department of Orthopedics, Peking University First Hospital, Beijing 100034, China.; ^#^These authors contributed equally to this work.

**Keywords:** Osteosarcoma, single-cell RNA sequencing, chemotherapy resistance, tumor microenvironment, prognostic signature, neoadjuvant chemotherapy

## Abstract

**Aim:** Osteosarcoma remains aggressive with poor prognosis, particularly in chemotherapy-resistant cases. This study aimed to characterize transcriptional features of chemoresistant osteosarcoma cells, establish a prognostic resistance signature, and identify therapeutic vulnerabilities.

**Methods:** Single-cell RNA sequencing (scRNA-seq) was performed on paired pre- and post-neoadjuvant chemotherapy (NAC) specimens from three patients (6 samples; 16,272 cells). Resistance trajectories were reconstructed using Monocle 3 pseudotime analysis. A nine-gene resistance score was validated in the Peking University People’s Hospital (PKPH) bulk RNA-seq cohort (*n* = 70) and Therapeutically Applicable Research to Generate Effective Treatments (TARGET) database (*n* = 87), with drug sensitivities predicted via oncoPredict.

**Results:** Chemotherapy reduced the malignant cell fraction but triggered expansion of cancer-associated fibroblasts and endothelial cells, creating a stromal-dominant, immune-sparse residual niche. Surviving tumor cells upregulated a nine-gene module along the resistance trajectory: *KCNMA1*, *KIF21A*, *MIR181A1HG*, *RPS27*, *PDPN*, *ADIRF*, *PRELP*, *PHEX*, and *COL9A2*. In an independent unpaired scRNA-seq cohort (two pre- and three post-chemotherapy samples), this signature remained associated with features of chemotherapy resistance. Higher scores correlated with poorer histopathologic response (r = -0.35, *P* = 0.006) and shorter progression-free survival [PKPH: hazard ratio (HR) = 2.4, 95% confidence interval (CI) 1.2-4.8, *P* = 0.01; TARGET: HR = 2.1, 95%CI 1.1-4.0, *P* = 0.02]. Of 198 compounds screened, only Pictilisib, a phosphoinositide 3-kinase (PI3K) inhibitor, showed lower predicted IC50 in the high-score subset across both datasets. However, the paired discovery cohort warrant further validation.

**Conclusion:** Our paired scRNA-seq approach identifies a nine-gene signature linking pre-treatment tumor biology to NAC response and outcome. The enhanced Pictilisib sensitivity in chemoresistant tumors positions PI3K blockade as a strategy meriting prospective testing in refractory osteosarcoma.

## INTRODUCTION

Osteosarcoma is the most common primary bone malignancy affecting children and adolescents^[1]^. However, despite four decades of clinical refinement, five-year survival has plateaued^[2]^. The treatment backbone remains neoadjuvant chemotherapy (NAC), typically methotrexate, doxorubicin, and cisplatin, followed by surgery^[3]^. Histopathologic response at resection stratifies responders from non-responders: tumors showing ≥ 90% necrosis qualify as good responders, and these patients experience 5-year survival near 70%. For patients with poor response to treatment, clinical outcomes become significantly worse. Local recurrence rates climb, metastatic spread becomes frequent, and 5-year survival in relapsed disease rarely exceeds 20%^[4,5]^. At the root of this therapeutic ceiling sits drug resistance, whether present from the outset or acquired during treatment.

A practical hurdle compounds the biological challenge: histopathologic response can only be assessed once the resection specimen arrives in pathology. Non-responders thus endure weeks of toxic, ultimately futile chemotherapy before their resistance becomes apparent. The mechanisms underlying this resistance remain incompletely understood. Work over the past decade has implicated ATP-binding cassette (ABC) transporter-mediated drug efflux, enhanced DNA repair, checkpoint evasion, apoptotic escape, and epithelial-mesenchymal transition (EMT)^[6,7]^. EMT appears particularly central because it promotes ABC transporter expression, activates phosphoinositide 3-kinase (PI3K)/protein kinase B (Akt) and mitogen-activated protein kinase (MAPK) survival pathways, and confers stem-like properties that blunt cytotoxic injury^[8,9]^. Bulk RNA sequencing (RNA-seq) studies have generated gene-expression classifiers for chemotherapy response, with immune- and microenvironment-derived transcripts proving especially informative^[10,11]^. However, bulk approaches hide cell-to-cell variation, averaging expression across heterogeneous populations and masking the dynamic transcriptional shifts and clonal complexity caused by treatment.

Single-cell RNA sequencing (scRNA-seq) resolves this limitation by capturing gene expression at single-cell resolution. In osteosarcoma, early scRNA-seq studies revealed considerable intratumoral heterogeneity alongside immunosuppressive microenvironmental features in advanced cases^[12]^. CD8+ T-cell density correlates with better chemotherapy response, and immune-gene panels achieve predictive areas under the curve (AUCs) of 0.72-0.83 across independent cohorts^[11]^. Comparing pre- and post-chemotherapy tissues has revealed therapy-driven remodeling: cancer-associated fibroblast (CAF) expansion, increased extracellular matrix (ECM) deposition, and reduced antitumor immune infiltrates and elevated stemness programs in residual malignant cells^[13]^. Functional screens have confirmed ATP-binding cassette subfamily B member 1 (ABCB1) and related efflux pumps as multidrug resistance mediators^[14]^, and podoplanin (PDPN) overexpression has emerged as a marker of lung metastasis and poor outcome, placing PDPN among candidate therapeutic targets^[15]^. Most of this single-cell work, however, rests on cross-sectional designs comparing different patients. This approach cannot track resistance trajectories or lineage dynamics within an individual tumor over time.

We sought to close this gap. Matched pre-NAC biopsy and post-NAC surgical specimens from three osteosarcoma patients underwent paired scRNA-seq, allowing us to follow treatment-induced cellular and molecular changes within each patient. Our objectives were fourfold: first, map cellular composition and gene expression changes across the NAC interval; second, identify transcriptomic features of residual chemoresistant populations; third, construct and validate a resistance signature against clinical endpoints in the Peking University People’s Hospital (PKPH) and Therapeutically Applicable Research to Generate Effective Treatments (TARGET) cohorts; and fourth, pinpoint candidate therapeutic vulnerabilities for chemorefractory disease. These data may ultimately facilitate earlier identification of resistant tumors and guide alternative treatment strategies for this difficult-to-treat population.

## METHODS

### Study design and patient cohorts

To dissect chemotherapy resistance at single-cell resolution, we collected paired pre- and post-NAC tumor specimens from three patients with osteosarcoma for scRNA-seq as the primary discovery cohort. In addition, five independent unpaired osteosarcoma samples, including two pre-NAC and three post-NAC specimens, were incorporated as a single-cell validation cohort to assess the reproducibility of the resistance-associated transcriptional program identified in the paired discovery cohort. These additional samples were processed and analyzed using the same experimental and computational workflow as the discovery cohort. For further validation, we assembled a retrospective cohort of 70 patients treated at PKPH with bulk RNA-seq data and clinical follow-up, and drew on the publicly available TARGET osteosarcoma dataset as an independent external cohort. PKPH patients were enrolled retrospectively if they had histologically confirmed osteosarcoma, adequate tumor tissue for RNA extraction, and complete clinical follow-up. All received standard MAP (methotrexate, doxorubicin, cisplatin) or AP (doxorubicin, cisplatin) neoadjuvant regimens between June 2012 and December 2023. Histologic response was assessed on resected specimens according to established histopathologic criteria. Consistent with the Huvos grading system and the convention adopted in major osteosarcoma studies, including EURAMOS-1, tumors with ≥ 90% necrosis were classified as good responders, whereas those with < 90% necrosis were classified as poor responders^[5]^. Representative H&E-stained sections illustrating different degrees of post-chemotherapy tumor necrosis are provided in Supplementary Figure 1. Overall survival (OS) was measured from diagnosis to death or last contact; progression-free survival (PFS), from diagnosis to progression or death. The Ethics Committee of Peking University People’s Hospital approved this study (No. 2024PHB432-001), which adhered to the Declaration of Helsinki. All participants or their guardians provided written informed consent.

### scRNA-seq

Three patients with paired pre-NAC biopsy and post-NAC surgical specimens were selected for scRNA-seq. Fresh tissue was placed in ice-cold RPMI-1640 with 2% fetal bovine serum (FBS) and transported to the laboratory within 30 min of collection. Enzymatic dissociation (Tumor Dissociation Kit, Miltenyi Biotec), filtration through 70 μm strainers, red blood cell lysis, and dead cell removal prepared single-cell suspensions. Trypan blue exclusion confirmed viability; samples below 80% viability were excluded. Libraries were prepared with the Chromium Single Cell 3’ Reagent Kit (10x Genomics) following manufacturer instructions. Roughly 10,000 cells per sample were loaded onto a Chromium Next GEM Chip G. Sequencing was performed on the Illumina NovaSeq 6000 with paired-end 150 bp reads, aiming for approximately 50,000 read pairs per cell.

### Single-cell data processing and analysis

Cell Ranger (10x Genomics) aligned raw reads to the GRCh38 reference genome and generated expression matrices. Quality control in Seurat v5.0 retained cells with 500 < nCount_RNA < 50,000, 200 < nFeature_RNA < 6,000, and mitochondrial fraction < 20%, leaving 16,272 cells across six samples. SCTransform normalized the data; Harmony v1.0 corrected batch effects. Principal component analysis (PCA) on the top 3,000 variable genes provided 30 principal components for downstream analysis. Uniform manifold approximation and projection (UMAP) embedding used n_neighbors = 30 and min_dist = 0.3. Louvain clustering at resolution = 0.8 identified 15 populations, annotated by canonical marker expression. InferCNV detected copy number variations (CNVs) from expression data, using non-malignant immune cells as a diploid reference. Cells in osteoblast-marker clusters that displayed large-scale CNVs were classified as malignant. To further characterize paired tumor microenvironment remodeling, we calculated sample-level module scores for T-cell exhaustion and tumor-associated macrophage (TAM) M1/M2 programs using annotated T cells and TAMs from each paired sample. We also performed a focused sender-receiver analysis among CAFs, TAMs, malignant cells, and endothelial cells using curated extracellular-matrix/adhesion, growth-factor, and immune-checkpoint ligand-receptor axes, and summarized the resulting interaction strengths at the paired sample level [Supplementary Figure 2]. Because pre-NAC CAF abundance was limited in informative paired samples, CAF subtype inference was not pursued in the paired comparison.

### Differential expression and trajectory analysis

Differentially expressed genes (DEGs) between pre-NAC and post-NAC tumor cells were identified via the Wilcoxon rank-sum test in Seurat. Genes with Bonferroni-adjusted *P* < 0.05 and |log2 fold change| > 1 were considered significant. Gene Ontology and Kyoto Encyclopedia of Genes and Genomes (KEGG) pathway enrichment analyses employed clusterProfiler v4.8.0. Gene Set Enrichment Analysis (GSEA) was conducted against Hallmark, KEGG, and Reactome gene sets from MSigDB with 10,000 permutations. Pseudotime trajectory analysis was performed in Monocle 3 v1.3.1. Pre-NAC tumor cells were set as the root state, and pseudotime values were computed to represent developmental progression toward chemotherapy-resistant phenotypes. Trajectory-associated genes were identified via Moran’s I test; genes with q-value < 0.01 were considered significantly trajectory-associated.

### Nine-gene resistance score

Drawing on trajectory analysis and differential expression results, a nine-gene chemotherapy resistance signature was established, comprising *KCNMA1*, *KIF21A*, *MIR181A1HG*, *RPS27*, *PDPN*, *ADIRF*, *PRELP*, *PHEX*, and *COL9A2*. These genes showed consistent upregulation in post-NAC tumor cells across all paired samples and significant association with pseudotime progression. The resistance score was computed as the sum of Z-score normalized expression values for the nine genes; patients were stratified into high-score and low-score groups using the median score as the cutoff.

### Survival analysis and drug sensitivity prediction

Survival curves were estimated by the Kaplan-Meier method and compared via the log-rank test. Univariate and multivariate Cox proportional hazards regression assessed prognostic significance. Hazard ratios (HRs) with 95% confidence intervals (CIs) were reported. Drug sensitivity was predicted using oncoPredict^[16]^, which applies ridge regression models trained on the Genomics of Drug Sensitivity in Cancer (GDSC) database to estimate IC50 values from bulk RNA-seq data. Predicted IC50 values were compared between resistance score groups via the Wilcoxon rank-sum test, with *P*-values adjusted for multiple testing by the Benjamini-Hochberg method. In the PKPH cohort, we additionally built multivariable Cox models for PFS and OS including the resistance score, age, sex, and histologic subtype, and visualized these models with forest plots and nomograms [Supplementary Figure 3A-D]. Model calibration at 12 and 24 months was assessed by comparing tertile-based mean predicted risk with Kaplan-Meier observed risk [Supplementary Figure 3E and F]. Model discrimination was further summarized by time-dependent AUC and bootstrap C-index [Supplementary Figure 3G], and 24-month decision-curve analysis compared the multivariable and score-only models [Supplementary Figure 3H]. Recurrence or metastasis were not included as baseline adjustment variables because, in this cohort, they mainly reflected follow-up events rather than pretreatment predictors. Histologic response was not included in the primary multivariable model because it is a post-treatment pathological variable closely related to the resistance score. To further contextualize the Pictilisib finding, we evaluated PI3K-related transcriptomic features in PKPH and TARGET using multiple scoring methods, including Hallmark PI3K-AKT-mechanistic target of rapamycin (mTOR) and a compact core PI3K-axis gene set, and examined their associations with tumor necrosis rate in PKPH [Supplementary Figure 4A-I].

### Statistical analysis

All statistical analyses were conducted in R v4.3.0. Data analysis and visualization were conducted using the R packages stats, Seurat, harmony, infercnv, monocle 3, clusterProfiler, survival, survminer, rms, timeROC, ggplot2, GSVA, and oncoPredict, as appropriate. Continuous variables were compared by Student’s *t*-test or Wilcoxon rank-sum test as appropriate. Categorical variables were analyzed by chi-square test or Fisher’s exact test. Multiple testing correction was applied using the Benjamini-Hochberg method. All tests were two-sided; *P* < 0.05 was the threshold for statistical significance.

## RESULTS

### Single-cell transcriptomic atlas of osteosarcoma before and after NAC

**Figure 1:**
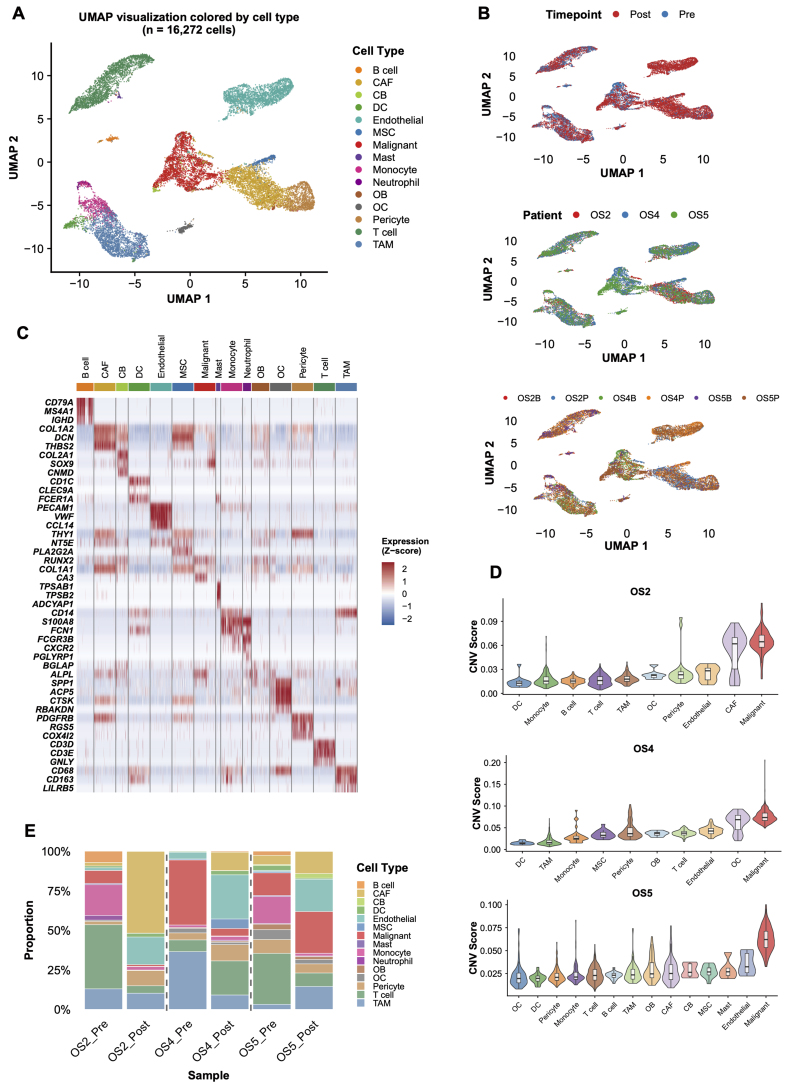
Single-cell transcriptomic atlas of osteosarcoma before and after NAC. (A) UMAP visualization of 16,272 cells from six samples (3 pre-NAC and 3 post-NAC) across three patients, colored by cell type. Fifteen distinct cell populations were identified through unsupervised clustering; (B) UMAP visualization colored by time point (pre-NAC, *n* = 3 samples; post-NAC, *n* = 3 samples), patient identity (OS2, OS4, OS5), and sample origin; (C) Heatmap showing the expression of canonical marker genes across the identified cell types; (D) InferCNV analysis distinguishing malignant cells (*n* = 2,942) from non-malignant populations based on inferred CNVs, using immune cells as diploid reference; (E) Stacked bar plot showing the proportions of each identified cell population in the six individual samples. NAC: Neoadjuvant chemotherapy; UMAP: uniform manifold approximation and projection; CNVs: copy number variations; CAF: cancer-associated fibroblast; CB: chondroblast; DC: dendritic cell; MSC: mesenchymal stem cells; OB: osteoblast; OC: osteoclast; TAM: tumor-associated macrophage.

Pre-NAC biopsies and post-NAC surgical specimens from three osteosarcoma patients underwent scRNA-seq to capture chemotherapy-driven cellular changes. After quality filtering, 16,272 cells from six samples entered analysis. Harmony batch correction and Louvain clustering (resolution 0.8) resolved 15 populations [Figure 1A and B]. InferCNV distinguished malignant cells from the broader osteoblast compartment via CNV; annotated clusters expressed expected canonical markers, and tumor cells showed substantially higher CNV burden than stromal or immune populations [Figure 1C and D]. Treatment transformed the cellular landscape. Pre-NAC, malignant cells dominated (26.22%), followed by T cells (21.77%) and TAMs (21.60%). Post-NAC, the hierarchy flipped: CAFs climbed to 25.21%, endothelial cells to 22.36%, and T cells dropped to 12.33% [Figure 1E]. The malignant fraction fell from 26.22% to 9.86%, consistent with cytotoxic tumor kill. Stromal compartments expanded in step: CAFs from 2.24% to 25.21%, endothelial cells from 2.64% to 22.36%, likely reflecting therapy-triggered stromal activation and angiogenic remodeling. Immune subsets shrank: T cells from 21.77% to 12.33%, TAMs from 21.60% to 11.00%, monocytes from 9.94% to 2.11%, a pattern fitting chemotherapy-induced immunosuppression. Paired functional-state analysis showed only modest post-treatment shifts in T-cell exhaustion and TAM polarization across the three cases, whereas the clearest remodeling was observed in stromal-tumor interaction patterns. In particular, CAF-to-malignant extracellular-matrix and adhesion-related axes, including COL1A1/COL1A2-ITGB1 and FN1-ITGB1, showed the most consistent increases after NAC [Supplementary Figure 2A-F].

### Chemotherapy-induced gene expression programs and identification of resistance-associated genes

**Figure 2:**
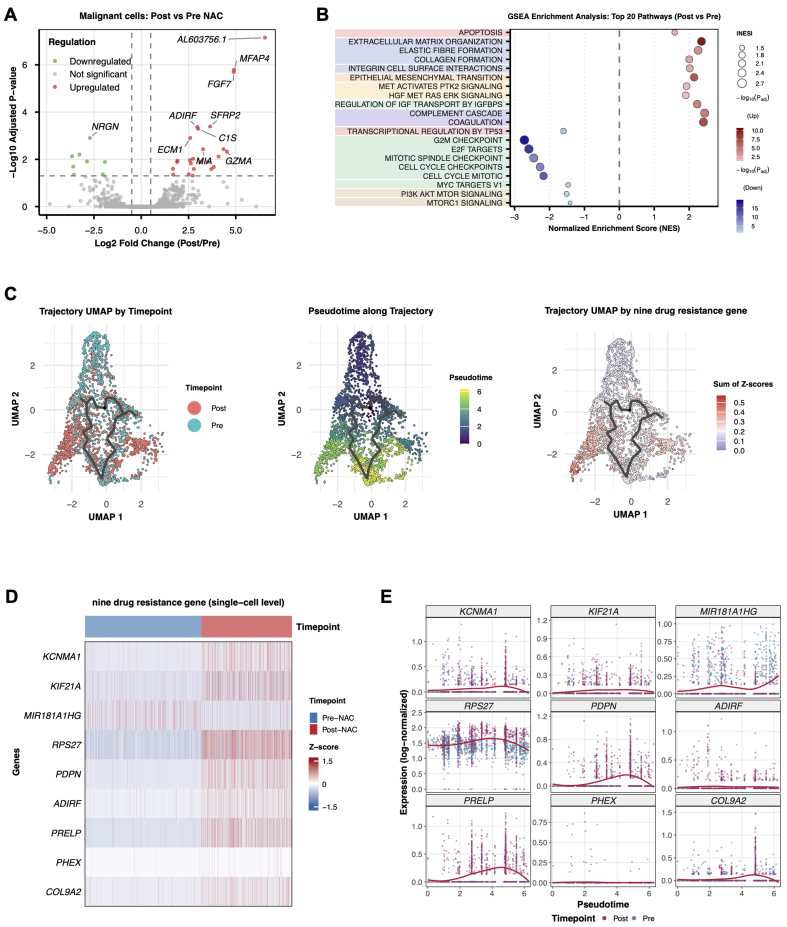
Chemotherapy-induced gene expression programs in tumor cells. (A) Volcano plot showing DEGs between pre-NAC and post-NAC tumor cells (*n* = 2,942 malignant cells total). Differential expression was assessed using a two-sided Wilcoxon rank-sum test with Bonferroni correction. Red dots indicate significantly upregulated genes; blue dots indicate significantly downregulated genes (adjusted *P* < 0.05, |log2FC| > 1); (B) GSEA showing differential pathway enrichment between post-NAC and pre-NAC tumor cells. Normalized enrichment scores and FDR q-values are indicated; (C) Pseudotime trajectory analysis of tumor cells using Monocle 3, reconstructing the developmental progression from pre-NAC (root state) to post-NAC resistant states. Cells are colored by timepoint (left), pseudotime value (middle), and the summed Z-score of the nine-gene resistance signature (right); (D) Heatmap displaying Z-score normalized expression levels of the nine resistance signature genes in pre-NAC (*n* = 1,847 cells) *vs.* post-NAC (*n* = 1,095 cells) tumor cells across all three patients; (E) Expression dynamics of the nine signature genes along the pseudotime trajectory, showing progressive upregulation toward resistant states. DEGs: Differentially expressed genes; NAC: neoadjuvant chemotherapy; GSEA: Gene Set Enrichment Analysis; FDR: false discovery rate.

Transcript-level comparison of pre- *vs.* post-NAC tumor cells revealed numerous DEGs [Figure 2A]. GSEA showed upregulation of ECM remodeling programs in surviving cells, including ECM organization, collagen deposition, and integrin-mediated adhesion. EMT ranked among the top enriched pathways, joined by hepatocyte growth factor (HGF)/MET proto-oncogene, receptor tyrosine kinase (MET) and insulin-like growth factor (IGF) signaling activation. Together, these changes point to enhanced invasive capacity and survival fitness in the residual population. Proliferation-linked modules moved in the opposite direction: tumor protein p53 (TP53)-mediated transcription, G2/mitotic (G2/M) checkpoint genes, E2F transcription factor (E2F) targets, MYC proto-oncogene (MYC) targets, and PI3K/AKT/mTOR signaling all fell [Figure 2B]. The picture that emerges is one of coordinated adaptation, with proliferative restraint paired against intensified matrix engagement and a shift toward a therapy-tolerant state. Monocle 3 pseudotime reconstruction tracked tumor cell evolution from drug-naïve to drug-tolerant states. Setting pre-NAC tumor cells as the root, pseudotime values traced progression toward chemoresistant phenotypes [Figure 2C]. The trajectory ran continuously from pre- to post-NAC malignant cells; genes significantly associated with pseudotime (Moran’s I test, q < 0.01) rose progressively along this continuum. Cross-referencing trajectory-correlated genes with differential expression data from matched samples yielded a core set consistently tied to chemotherapy-driven resistance. Nine genes, *KCNMA1*, *KIF21A*, *MIR181A1HG*, *RPS27*, *PDPN*, *ADIRF*, *PRELP*, *PHEX*, and *COL9A2*, showed reproducible upregulation across all paired samples and significant trajectory association [Figure 2D and E]. These transcripts formed the basis for a composite resistance score.

### An independent unpaired scRNA-seq cohort supports the reproducibility of the resistant malignant-cell program

**Figure 3:**
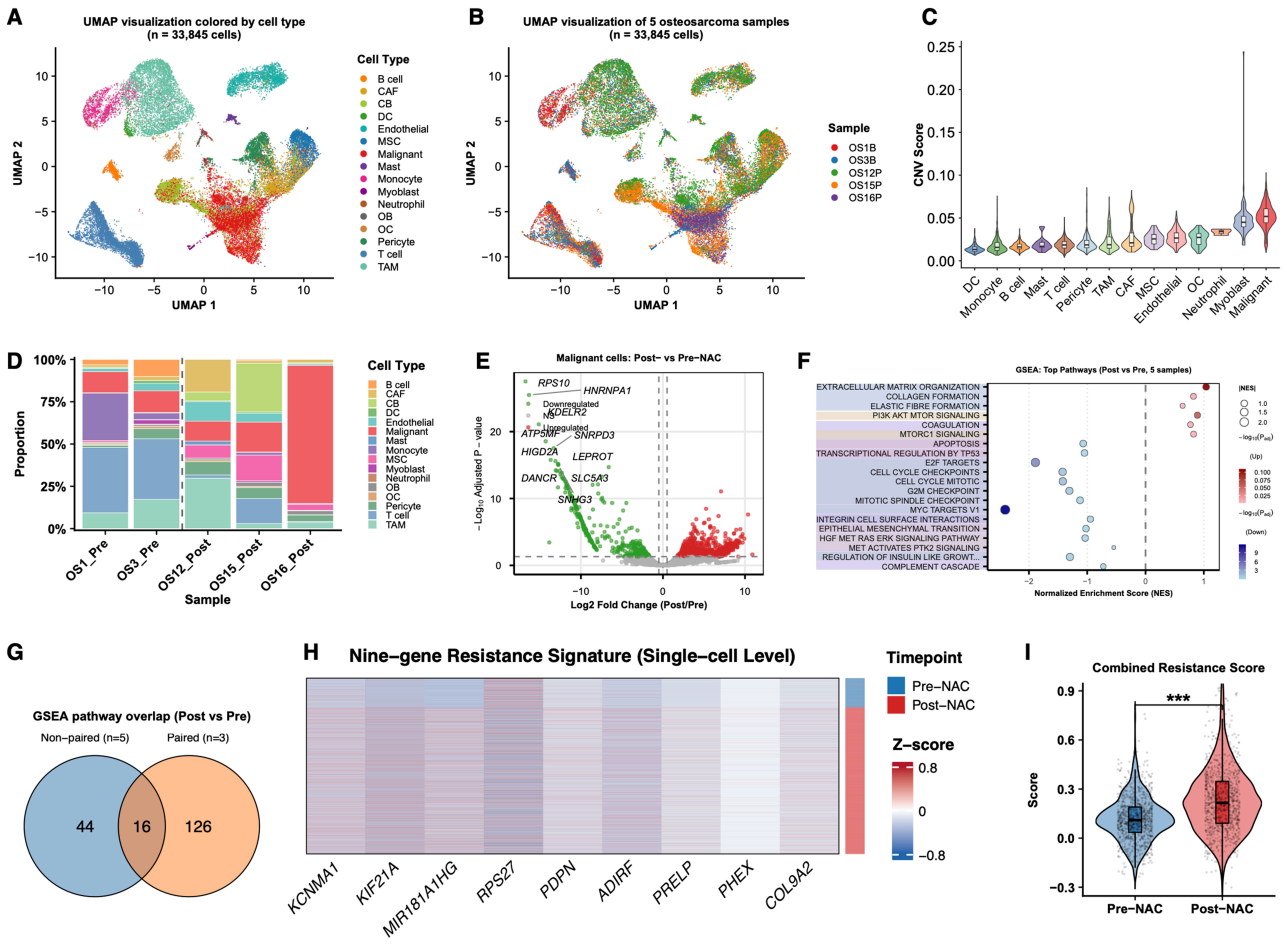
Independent unpaired scRNA-seq cohort supports the reproducibility of the resistance-associated malignant-cell program. (A) UMAP visualization of 33,845 single cells from five osteosarcoma samples, colored by annotated cell type; (B) UMAP visualization of the same cells colored by sample origin, including two pre-NAC samples (OS1B and OS3B) and three post-NAC samples (OS12P, OS15P, and OS16P); (C) Comparison of inferCNV scores across annotated cell populations, showing higher inferCNV signals in osteoblast-like malignant cells than in non-malignant microenvironmental cells; (D) Stacked bar plot showing the relative proportions of major cell populations across individual samples; (E) Exploratory differential expression analysis of malignant cells comparing post-NAC *vs.* pre-NAC samples; (F) Dot plot showing the top enriched pathways identified by GSEA in malignant cells from post-NAC *vs.* pre-NAC samples. Dot size indicates significance, and color indicates normalized enrichment score direction; (G) Overlap of significantly enriched pathways between the independent five-sample validation cohort and the paired discovery cohort; (H) Heatmap showing the expression of the nine resistance-associated genes in malignant cells, grouped by treatment status and sample origin; (I) Violin plot showing the combined resistance score calculated from the nine-gene signature in malignant cells from the pre-NAC and post-NAC groups. For (E), differential expression was assessed using a two-sided Wilcoxon rank-sum test. For (I), *P* values were calculated using a two-sided Wilcoxon rank-sum test; ^***^P < 0.001. scRNA-seq: Single-cell RNA sequencing; UMAP: uniform manifold approximation and projection; NAC: neoadjuvant chemotherapy; CNV: copy number variation; GSEA: Gene Set Enrichment Analysis; CAF: cancer-associated fibroblast; CB: chondroblast; DC: dendritic cell; MSC: mesenchymal stem cells; OB: osteoblast; OC: osteoclast; TAM: tumor-associated macrophage.

To further assess the reproducibility of the resistance-associated program identified in the paired discovery cohort, we analyzed an independent unpaired scRNA-seq cohort comprising five osteosarcoma samples, including two pre-NAC and three post-NAC samples. A total of 33,845 cells were retained after quality control and classified into 14 major cell populations [Figure 3A and B]. InferCNV analysis provided orthogonal support for malignant-cell annotation by showing higher inferCNV scores in osteoblast-like malignant cells than in non-malignant cell populations [Figure 3C]. Cell composition analysis revealed marked heterogeneity across samples and treatment groups [Figure 3D]. In malignant cells, exploratory differential expression and pathway enrichment analyses showed that post-NAC samples displayed resistance-associated transcriptional features that overlapped with those observed in the paired discovery cohort [Figure 3E-G]. Moreover, the nine-gene resistance signature was more highly expressed in post-NAC malignant cells, and the combined resistance score was significantly increased in the post-NAC group [Figure 3H and I]. Together, these findings support the reproducibility of the resistant malignant-cell program in an independent single-cell dataset.

### Validation of the nine-gene resistance signature in bulk RNA-seq cohorts

**Figure 4:**
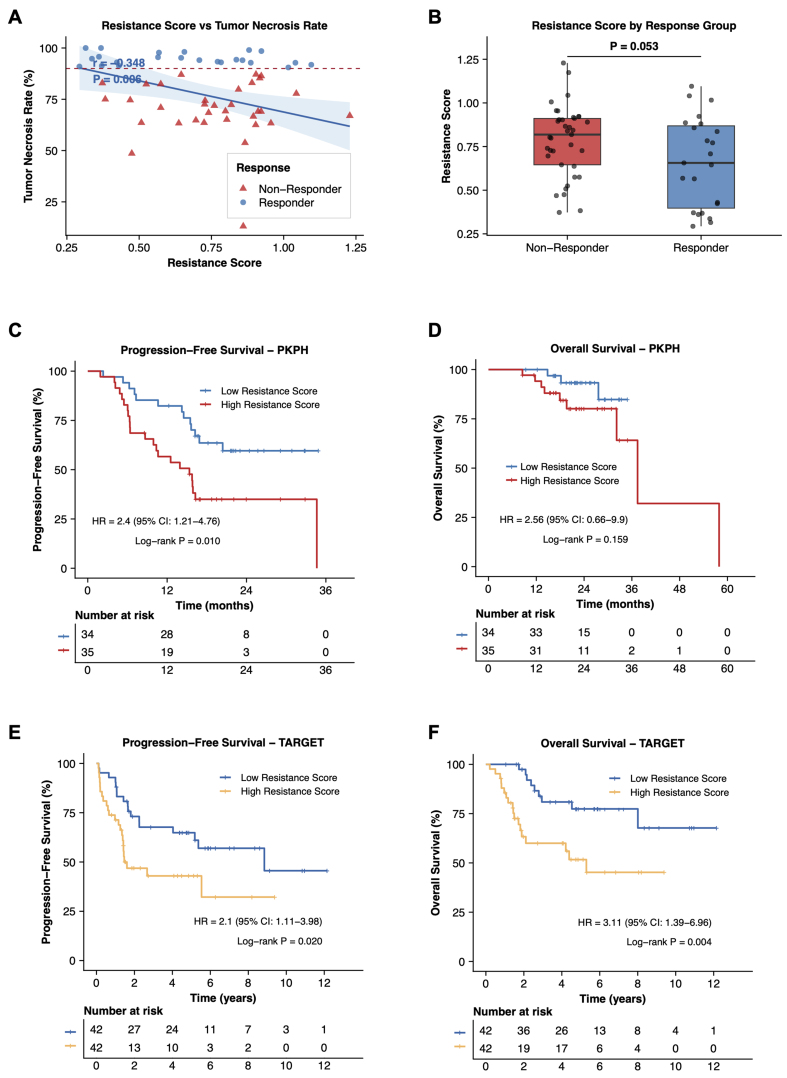
Identification and validation of the nine-gene resistance signature. (A) Correlation between resistance score and tumor necrosis rate in the PKPH cohort (*n* = 70). Pearson correlation coefficient (r) and *P*-value are shown; (B) Box plot comparing resistance scores between responders (tumor necrosis rate ≥ 90%) and non-responders (< 90%) in the PKPH cohort. *P*-value from Wilcoxon rank-sum test; (C) Kaplan-Meier curves for PFS in the PKPH cohort stratified by high *vs.* low resistance score using median cutoff. HR, 95%CI, and log-rank *P* value are indicated; (D) Kaplan-Meier curves for OS in the PKPH cohort; (E) Kaplan-Meier curves for PFS in the TARGET cohort stratified by resistance score; (F) Kaplan-Meier curves for OS in the TARGET cohort. For (A), the correlation was assessed using a two-sided Pearson correlation test; for (B), group comparison was performed using a two-sided Wilcoxon rank-sum test; for (C-F), survival differences were assessed using two-sided log-rank tests. PKPH: Peking University People’s Hospital; PFS: progression-free survival; HR: hazard ratio; CI: confidence interval; OS: overall survival; TARGET: Therapeutically Applicable Research to Generate Effective Treatments.

We next tested the clinical relevance of the nine-gene signature in bulk RNA-seq data from 70 osteosarcoma patients (PKPH cohort) who received standard NAC followed by resection. Table 1 details clinical characteristics. Patients split at the median score showed no significant baseline differences in age, histologic subtype, or tumor site between groups. The resistance score inversely correlated with tumor necrosis rate (r = -0.35, *P* = 0.006; Figure 4A). Patients achieving ≥ 90% necrosis (good responders) carried lower scores than non-responders (*P* = 0.05; Figure 4B). High-score patients had shorter PFS (HR = 2.4, 95%CI: 1.2-4.8, log-rank *P* = 0.01) and a non-significant trend toward shorter OS (HR = 2.6, 95%CI: 0.7-9.9, log-rank *P* = 0.16) compared to the low-score group [Figure 4C and D]. The TARGET cohort corroborated these findings: elevated scores associated with worse PFS (HR = 2.1, 95%CI: 1.1-4.0, log-rank *P* = 0.02) and OS (HR = 3.1, 95%CI: 1.4-7.0, log-rank *P* = 0.004; Figure 4E and F). Additional sensitivity analyses using different score cutoffs in the bulk RNA-seq cohorts showed that the associations of the nine-gene score with tumor necrosis and survival outcomes remained generally stable [Supplementary Figure 5]. In sum, the nine-gene score inversely reflects chemotherapy sensitivity as indexed by tumor necrosis and stratifies patient outcome in osteosarcoma, although the OS association in the PKPH cohort did not reach statistical significance. To present the prognostic modeling more completely, we further analyzed the PKPH cohort with multivariable Cox models including age, sex, and histologic subtype. The resistance score retained a consistent adverse association direction in the adjusted PFS model, whereas the adjusted OS model was interpreted cautiously because of the limited number of OS events. Nomograms, 12- and 24-month calibration plots, time-dependent discrimination curves, bootstrap C-index summaries, and 24-month decision-curve analysis are provided in Supplementary Figure 3A-H.

**Table 1:** Clinical characteristics of osteosarcoma patients stratified by resistance score

**Characteristic**	**All patients**	**High resistance**	**Low resistance**	***P* value**
No. of patients	70	35	35	
Age, years, median (IQR)	14.0 (11.2-16.0)	15.0 (12.0-16.5)	13.0 (11.0-15.0)	0.22
Sex, *n* (%)				0.47
Male	32 (45.7%)	14 (40.0%)	18 (51.4%)	
Female	38 (54.3%)	21 (60.0%)	17 (48.6%)	
Primary tumor site, *n* (%)				0.25
Femur	44 (62.9%)	19 (54.3%)	25 (71.4%)	
Tibia	13 (18.6%)	9 (25.7%)	4 (11.4%)	
Humerus	12 (17.1%)	7 (20.0%)	5 (14.3%)	
Fibula	1 (1.4%)	0 (0.0%)	1 (2.9%)	
Histologic subtype, *n* (%)				0.49
Conventional osteosarcoma	68 (97.1%)	33 (94.3%)	35 (100.0%)	
Other subtypes	2 (2.9%)	2 (5.7%)	0 (0.0%)	
Targeted therapy, *n* (%)				0.24
Yes	15 (21.4%)	10 (28.6%)	5 (14.3%)	
No	55 (78.6%)	25 (71.4%)	30 (85.7%)	
Immunotherapy, *n* (%)				0.26
Yes	17 (24.3%)	11 (31.4%)	6 (17.1%)	
No	53 (75.7%)	24 (68.6%)	29 (82.9%)	
Recurrence, *n* (%)				0.28
Yes	19 (27.1%)	12 (34.3%)	7 (20.0%)	
No	51 (72.9%)	23 (65.7%)	28 (80.0%)	
Metastasis, *n* (%)				0.06
Yes	35 (50.0%)	22 (62.9%)	13 (37.1%)	
No	35 (50.0%)	13 (37.1%)	22 (62.9%)	
Death, *n* (%)				0.06
Yes	13 (18.6%)	10 (28.6%)	3 (8.6%)	
No	57 (81.4%)	25 (71.4%)	32 (91.4%)	

*P* values were calculated using the Wilcoxon rank-sum test for continuous variables and the chi-square test or Fisher’s exact test for categorical variables. IQR: Interquartile range.

### Drug sensitivity prediction identifies potential therapeutic targets for chemotherapy-resistant osteosarcoma

**Figure 5:**
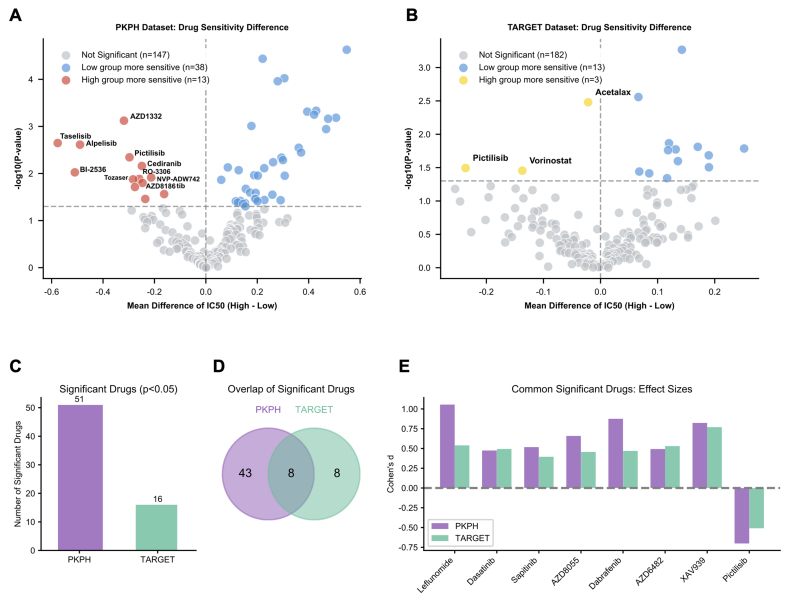
Drug sensitivity prediction identifies PI3K inhibition as a therapeutic vulnerability. (A) Volcano plot displaying predicted drug sensitivity differences (log2 fold change of IC50) between resistance score-high (*n* = 35) and score-low (*n* = 35) groups in the PKPH cohort. Drugs with significantly different sensitivities (FDR-adjusted *P* < 0.05) are highlighted; positive values indicate higher IC50 (resistance) in the high-score group; (B) Volcano plot displaying drug sensitivity differences between resistance score groups in the TARGET cohort (high, *n* = 43; low, *n* = 44); (C) Bar chart showing the number of drugs with significantly different predicted sensitivities in each cohort (PKPH: 51 drugs; TARGET: 16 drugs); (D) Venn diagram illustrating the overlap of drugs with significant sensitivity differences between cohorts, identifying 8 concordant drugs; (E) Bar chart showing effect sizes (Cohen’s d) for the eight drugs with concordant significant sensitivity differences. Notably, Pictilisib (PI3K inhibitor) is the only drug showing enhanced sensitivity (negative Cohen’s d, lower IC50) in the high-resistance-score group in both cohorts. For (A and B), drug sensitivity differences were assessed using two-sided Wilcoxon rank-sum tests with Benjamini-Hochberg correction. PI3K: Phosphoinositide 3-kinase; PKPH: Peking University People’s Hospital; FDR: false discovery rate; TARGET: Therapeutically Applicable Research to Generate Effective Treatments.

Could alternative agents benefit patients with high resistance scores? Using oncoPredict, we estimated drug sensitivities stratified by score. In the PKPH cohort, comparing predicted IC50 values between high- and low-score groups flagged 51 drugs with significantly different sensitivities [false discovery rate (FDR)-adjusted *P* < 0.05; Figure 5A]. Most showed elevated IC50, indicating reduced sensitivity, in the high-score group, as expected for a chemorefractory subset. The TARGET cohort yielded 16 drugs with differential sensitivities between strata [Figure 5B]; again, the majority showed higher IC50 in high-score patients. Chemoresistance-high osteosarcoma thus appears broadly multidrug resistant. Intersecting differentially sensitive drugs across cohorts identified eight agents with concordant patterns [Figure 5C and D]. One outlier stood out: Pictilisib, a PI3K inhibitor, displayed enhanced sensitivity in the high-score group in both datasets [Figure 5E], the only agent to do so. This finding raises the possibility that PI3K pathway blockade may offer a therapeutic opportunity for chemorefractory osteosarcoma and provides a rationale for biomarker-guided patient selection. To further relate this vulnerability to PI3K-related transcriptomic features, we performed multimethod PI3K scoring in PKPH and TARGET. Among the evaluated metrics, a compact core PI3K-axis mean Z score showed the most consistent positive association with the resistance score across cohorts (PKPH: r = 0.36, *P* = 0.002; TARGET: r = 0.23, *P* = 0.031), and PIK3CA expression was positively associated in PKPH with a similar trend in TARGET [Supplementary Figure 4A-G]. By contrast, correlations between PI3K-related transcriptomic features and tumor necrosis rate in PKPH were weak and not statistically significant [Supplementary Figure 4H-I]. These results support an association between the resistance program and PI3K-related transcriptomic features, but do not by themselves establish uniform pathway activation.

## DISCUSSION

By profiling matched pre- and post-NAC osteosarcoma tissues from the same patients at single-cell resolution, we traced chemotherapy resistance trajectories in a way cross-sectional comparisons cannot. NAC remodeled the microenvironment: CAFs and endothelial cells expanded while immune populations receded. Residual tumor cells converged on a drug-tolerant program characterized by EMT and ECM gene induction, coupled with dampened proliferative signaling. From differential expression and pseudotime analyses, we distilled a nine-gene signature that inversely correlated with tumor necrosis and stratified patient prognosis in two independent cohorts. Pharmacogenomic modeling flagged Pictilisib, a PI3K inhibitor, as the only agent with heightened predicted activity against the chemoresistant subset.

The stromal shift merits closer examination. Chemotherapy killed tumor cells, yet CAFs and endothelial populations expanded in parallel; T cells and TAMs dwindled. One reading of these data is that therapy-induced tissue damage triggers wound-healing programs, producing an ECM-dense, hypervascularized, but immunologically sparse environment that shields surviving tumor cells. CAF expansion and intensified ECM remodeling may erect both physical and biochemical barriers: limited drug penetration, restricted immune access, and paracrine survival signals such as HGF^[17]^. Studies in osteosarcoma and other solid tumors point to CAF-derived ECM as a driver of drug resistance, angiogenesis, and immune exclusion^[11,18]^, the immune-hot *vs.* immune-cold dichotomy now figures prominently in treatment response discussions^[19]^. Because our design tracks individual patients through therapy, it shows directly that these compositional shifts arise from treatment rather than baseline inter-patient variation, extending prior cross-sectional observations^[11,13]^. Whether conventional chemotherapy inadvertently fortifies a stromal-dominated, immune-excluded sanctuary for resistant clones warrants further mechanistic inquiry.

Focusing on surviving tumor cells, we observed coordinated upregulation of EMT and ECM remodeling modules (collagen biosynthesis, integrin-dependent adhesion, matrix-interaction networks), alongside HGF/MET and IGF signaling. Proliferation-linked programs receded: TP53-mediated transcription, G2/M checkpoint genes, E2F and MYC targets, and PI3K/AKT/mTOR signaling all fell. This pattern echoes the drug-tolerant persister phenotype described in other cancers, where cells trade proliferative drive for survival capacity and stronger matrix attachment^[20]^. Several mechanistic threads link this EMT-ECM phenotype to chemoresistance: integrin-ECM interactions fire pro-survival kinase cascades; quiescent or slow-cycling cells escape cytotoxic agents that preferentially kill dividing cells; mesenchymal conversion amplifies stemness and migratory capacity^[8,21]^. Dense collagen deposition physically impedes drug distribution while feeding back through integrins and growth-factor receptors to reinforce the resistant compartment^[22,23]^. The HGF/MET enrichment we observed mirrors stromal-mediated resistance mechanisms documented in epithelial malignancies^[24]^, and the IGF pathway activation aligns with earlier reports implicating insulin-like growth factor 1 receptor (IGF1R) in osteosarcoma survival and chemoresistance^[25]^. These pathway shifts suggest druggable nodes, though functional experiments are needed to confirm causality.

Combining pseudotime trajectory analysis with differential expression, we traced a continuum from drug-sensitive to drug-resistant states and extracted a nine-gene signature that rose progressively along this axis, accumulated in post-NAC cells, and was consistently elevated across patients. Notably, the progressive increase of these genes along pseudotime suggests that they represent a treatment-associated adaptive program rather than a static marker set. Functionally, these genes appear to cluster around several resistance-related modules. *PDPN*, *PRELP*, and *COL9A2* participate in ECM and cell-surface remodeling, which may facilitate stromal interaction and support the formation of a protective residual niche after chemotherapy^[26]^. *KIF21A* and *KCNMA1* are cytoskeletal and vesicular transport mediators that may influence motility and membrane physiology^[27]^, potentially contributing to cellular plasticity and stress adaptation under therapeutic pressure, and may also be relevant to multidrug resistance through altered transport-related and ion channel-associated adaptive responses^[28,29]^. *ADIRF* and *RPS27*, metabolic and ribosomal regulators, may reflect altered protein synthesis and metabolic rewiring^[30]^, both of which are commonly associated with drug-tolerant states. *PHEX* relates to bone-specific mineralization, and *MIR181A1HG* may exert post-transcriptional control, suggesting possible links to osteogenic reprogramming and transcriptional adaptability during resistance evolution, while also raising the possibility of multidrug resistance (MDR)-related post-transcriptional adaptation, given the reported association of the miR-181 family with chemotherapy response and resistance pathways^[31]^. Taken together, this gene set may capture a coordinated chemoresistance-associated program shaped by both tumor-intrinsic adaptation and microenvironmental remodeling**.** These interpretations should be viewed as biologically plausible hypotheses derived from trajectory-associated expression dynamics rather than functionally validated mechanisms. Its derivation from within-patient resistance trajectories distinguishes it from cross-sectional signatures and may enhance its biological interpretability.

To enhance the robustness of our findings, we additionally analyzed an independent unpaired scRNA-seq cohort consisting of two pre-NAC and three post-NAC osteosarcoma samples. Although these samples were not patient-matched and therefore were not used for longitudinal inference, they provided an orthogonal single-cell validation dataset. Importantly, this validation cohort recapitulated the malignant-cell identity, resistance-associated pathway enrichment, and elevated nine-gene resistance score observed in the paired discovery cohort. These findings support the reproducibility of the identified resistant malignant-cell program across independent osteosarcoma samples. Nevertheless, because this validation cohort was unpaired and limited in sample size, these results should be interpreted as supportive rather than definitive evidence, and larger prospective cohorts will be needed to further assess inter-patient heterogeneity and treatment-associated transcriptional evolution.

External validation in the PKPH and TARGET cohorts confirmed the prognostic value of this signature; its inverse correlation with tumor necrosis indicates that it captures biology relevant to chemotherapy response. Notably, in the PKPH cohort, the association was strongest for tumor necrosis and PFS, while only a non-significant trend was observed for OS; by contrast, both PFS and OS were significantly associated with the score in the TARGET cohort. Earlier osteosarcoma studies have proposed bulk transcriptomic or immune-related signatures to forecast NAC response or survival^[10,11,32]^, but these typically derive from cross-sectional comparisons without explicitly encoding a therapy-induced evolutionary trajectory. Our signature, by contrast, reflects the within-patient transition from drug-naïve to drug-tolerant states, which may enhance its mechanistic interpretability.

From a clinical standpoint, these findings speak to an unmet need. Oncologists treating osteosarcoma currently lack early predictors of NAC response; tumor necrosis is only assessable after resection, leaving patients destined for failure to endure weeks of intensive chemotherapy with limited benefit and substantial toxicity. The inverse relationship between our nine-gene score and necrosis rate suggests that pre-treatment biopsy profiling might identify likely non-responders before surgery, creating an opportunity for treatment modification or intensification. Prospective validation will be essential before clinical adoption. Beyond risk stratification, our pharmacogenomic analysis singled out Pictilisib as the sole PI3K inhibitor with lower predicted IC50 in the high-score, chemoresistant subset in both cohorts. Preclinical data already support PI3K pathway vulnerability in osteosarcoma models, and early-phase PI3K inhibitor trials in bone sarcomas are ongoing. Testing Pictilisib or similar compounds in molecularly selected poor responders is a logical next step.

Several limitations warrant acknowledgment. Our original paired single-cell analysis involved only three patients; while this design enables within-patient tracking, it cannot capture the full genomic and microenvironmental diversity of osteosarcoma. Although we incorporated five additional unpaired osteosarcoma samples to support the main findings, the overall single-cell cohort remains limited in size, and larger multi-center studies, ideally with additional paired pre-/post-NAC samples, will be needed for further validation. Discovery and single-cell samples came from one institution with predominantly East Asian patients; TARGET validation partially addresses generalizability, but prospective testing in ethnically diverse populations remains necessary. The signature was defined and validated at the transcriptomic level without protein-level confirmation; future studies should assess concordance by immunohistochemistry and develop clinically deployable assays. OncoPredict predictions rely on *in vitro* cell-line pharmacogenomic datasets that do not fully recapitulate patient-specific pharmacokinetics, clonal architecture, or stromal interactions. Therefore, the drug-sensitivity results reported here should be interpreted as computational predictions and will require further validation through dedicated *in vitro* and *in vivo* drug-response studies.

### Conclusion

Paired scRNA-seq of matched pre- and post-NAC osteosarcoma specimens illuminates how chemotherapy reshapes the microenvironment and how tumor cells transition toward drug tolerance. A nine-gene resistance signature emerged from these within-patient trajectories and, in two independent cohorts, correlated with tumor necrosis and patient prognosis, providing a potential tool for early response assessment and risk stratification. The selective Pictilisib sensitivity predicted in chemoresistant tumors points to PI3K inhibition as a biomarker-guided strategy worthy of prospective testing. Together, these data connect single-cell biology to clinical outcomes and support precision-medicine approaches in osteosarcoma management.

## DECLARATIONS

### Availability of data and materials

The single-cell RNA sequencing data generated in this study has been deposited in the Genome Sequence Archive of the Beijing Institute of Genomics (BIG) Data Center, BIG, Chinese Academy of Sciences, under accession code HRA013407, which are publicly accessible at https://ngdc.cncb.ac.cn/gsa-human/. The TARGET-OS dataset is publicly available at https://ocg.cancer.gov/programs/target. The PKPH-OS dataset reported in this study have been deposited in the Gene Expression Omnibus (GEO) database of the National Center for Biotechnology Information (NCBI) under accession number GSE317512.

### AI and AI-assisted tools statement

During the preparation of this manuscript, the AI tool GPT (version 5.2, released 2025-12-11) was used solely for language editing. The tool did not influence the study design, data collection, analysis, interpretation, or the scientific content of the work. All authors take full responsibility for the accuracy, integrity, and final content of the manuscript.

### Copyright

© The Author(s) 2026.

## References

[1] Mirabello L, Troisi RJ, Savage SA (2009). Osteosarcoma incidence and survival rates from 1973 to 2004: data from the Surveillance, Epidemiology, and End Results Program. Cancer.

[2] Bielack SS, Kempf-Bielack B, Delling G (2002). Prognostic factors in high-grade osteosarcoma of the extremities or trunk: an analysis of 1,702 patients treated on neoadjuvant cooperative osteosarcoma study group protocols. J Clin Oncol.

[3] (2003). Kager L, Zoubek A, Pötschger U, et al.; Cooperative German-Austrian-Swiss Osteosarcoma Study Group. Primary metastatic osteosarcoma: presentation and outcome of patients treated on neoadjuvant Cooperative Osteosarcoma Study Group protocols. J Clin Oncol.

[4] Bacci G, Mercuri M, Longhi A (2005). Grade of chemotherapy-induced necrosis as a predictor of local and systemic control in 881 patients with non-metastatic osteosarcoma of the extremities treated with neoadjuvant chemotherapy in a single institution. Eur J Cancer.

[5] (2015). Whelan JS, Bielack SS, Marina N, et al.; EURAMOS collaborators. EURAMOS-1, an international randomised study for osteosarcoma: results from pre-randomisation treatment. Ann Oncol.

[6] Garcia-Ortega DY, Cabrera-Nieto SA, Caro-Sánchez HS, Cruz-Ramos M (2022). An overview of resistance to chemotherapy in osteosarcoma and future perspectives. Cancer Drug Resist.

[7] Lilienthal I, Herold N (2020). Targeting molecular mechanisms underlying treatment efficacy and resistance in osteosarcoma: a review of current and future strategies. Int J Mol Sci.

[8] Du B, Shim JS (2016). Targeting epithelial-mesenchymal transition (EMT) to overcome drug resistance in cancer. Molecules.

[9] Sannino G, Marchetto A, Kirchner T, Grünewald TGP (2017). Epithelial-to-mesenchymal and mesenchymal-to-epithelial transition in mesenchymal tumors: a paradox in sarcomas?. Cancer Res.

[10] Zeng Z, Li W, Zhang D (2022). Development of a chemoresistant risk scoring model for prechemotherapy osteosarcoma using single-cell sequencing. Front Oncol.

[11] He L, Yang H, Huang J (2021). The tumor immune microenvironment and immune-related signature predict the chemotherapy response in patients with osteosarcoma. BMC Cancer.

[12] Zhou Y, Yang D, Yang Q (2020). Single-cell RNA landscape of intratumoral heterogeneity and immunosuppressive microenvironment in advanced osteosarcoma. Nat Commun.

[13] Zheng X, Wu W, Zhao Z, Zhang X, Yu S (2024). Single-cell transcriptomic insights into chemotherapy-induced remodeling of the osteosarcoma tumor microenvironment. J Cancer Res Clin Oncol.

[14] Liu T, Li Z, Zhang Q (2016). Targeting ABCB1 (MDR1) in multi-drug resistant osteosarcoma cells using the CRISPR-Cas9 system to reverse drug resistance. Oncotarget.

[15] Takemoto A, Takagi S, Ukaji T (2022). Targeting podoplanin for the treatment of osteosarcoma. Clin Cancer Res.

[16] Maeser D, Gruener RF, Huang RS (2021). oncoPredict: an R package for predicting in vivo or cancer patient drug response and biomarkers from cell line screening data. Brief Bioinform.

[17] Feng B, Wu J, Shen B, Jiang F, Feng J (2022). Cancer-associated fibroblasts and resistance to anticancer therapies: status, mechanisms, and countermeasures. Cancer Cell Int.

[18] Straussman R, Morikawa T, Shee K (2012). Tumour micro-environment elicits innate resistance to RAF inhibitors through HGF secretion. Nature.

[19] Galon J, Bruni D (2019). Approaches to treat immune hot, altered and cold tumours with combination immunotherapies. Nat Rev Drug Discov.

[20] Sharma SV, Lee DY, Li B (2010). A chromatin-mediated reversible drug-tolerant state in cancer cell subpopulations. Cell.

[21] Kesh K, Gupta VK, Durden B (2020). Therapy resistance, cancer stem cells and ECM in cancer: the matrix reloaded. Cancers.

[22] Huang J, Zhang L, Wan D (2021). Extracellular matrix and its therapeutic potential for cancer treatment. Signal Transduct Target Ther.

[23] Piersma B, Hayward MK, Weaver VM (2020). Fibrosis and cancer: a strained relationship. Biochim Biophys Acta Rev Cancer.

[24] Wilson TR, Fridlyand J, Yan Y (2012). Widespread potential for growth-factor-driven resistance to anticancer kinase inhibitors. Nature.

[25] Tzanakakis GN, Giatagana EM, Berdiaki A (2021). The role of IGF/IGF-IR-signaling and extracellular matrix effectors in bone sarcoma pathogenesis. Cancers.

[26] Astarita JL, Acton SE, Turley SJ (2012). Podoplanin: emerging functions in development, the immune system, and cancer. Front Immunol.

[27] van der Vaart B, van Riel WE, Doodhi H (2013). CFEOM1-associated kinesin KIF21A is a cortical microtubule growth inhibitor. Dev Cell.

[28] Xia C, Liu C, Ren S, Cai Y, Zhang Q, Xia C (2023). Potassium channels, tumorigenesis and targeted drugs. Biomed Pharmacother.

[29] Groth-Pedersen L, Aits S, Corcelle-Termeau E, Petersen NH, Nylandsted J, Jäättelä M (2012). Identification of cytoskeleton-associated proteins essential for lysosomal stability and survival of human cancer cells. PLoS One.

[30] Warner JR, McIntosh KB (2009). How common are extraribosomal functions of ribosomal proteins?. Mol Cell.

[31] Jiao X, Zhao L, Ma M (2013). MiR-181a enhances drug sensitivity in mitoxantone-resistant breast cancer cells by targeting breast cancer resistance protein (BCRP/ABCG2). Breast Cancer Res Treat.

[32] Man TK, Chintagumpala M, Visvanathan J (2005). Expression profiles of osteosarcoma that can predict response to chemotherapy. Cancer Res.

