# Supplementary material for: Paired single cell analysis reveals chemotherapy resistance in osteosarcoma
Source: Cancer Drug Resist. 2026 Apr 21;9:13. doi: 10.20517/cdr.2026.09 (PMC13174192; doi:10.20517/cdr.2026.09)
Supplement: Supplementary file 1 [file cdr-9-13-SupplementaryMaterials.pdf]

Supplementary Materials

Paired single cell analysis reveals chemotherapy resistance in osteosarcoma

Li Hu[1,#], Yaxin Zhang[1,#], Boyang Wang[2,#], Qian Liu[1], Feiyang Qi[2], Huimin Liu[3], Qinghua Li[4], Zhiqing Zhao[5], Haijie Liang[2], Xingyu Liu[2], Zhiye Du[2], Jichuan Wang[2]

[1]Familial & Hereditary Cancer Center, Peking University Cancer Hospital & Institute, Key Laboratory of Carcinogenesis and Translational Research (Ministry of Education), Beijing 100142, China.
[2]Musculoskeletal Tumor Center, Beijing Key Laboratory for Musculoskeletal Tumors, Peking University People's Hospital, Beijing 100044, China.
[3]Multidisciplinary Diagnosis and Treatment Center for Bone Tumors, Peking University Shougang Hospital, Beijing 100144, China.
[4]Department of Biochemistry and Molecular Biology, School of Basic Medical Sciences, Peking University International Cancer Institute, Peking University Health Science Center, Beijing 100191, China.
[5]Department of Orthopedics, Peking University First Hospital, Beijing 100034, China.
[#]These authors contributed equally to this work.

Correspondence to: Dr. Jichuan Wang, Musculoskeletal Tumor Center, Beijing Key Laboratory for Musculoskeletal Tumors, Peking University People's Hospital, Beijing 100044, China. E-mail: jcwang@pku.edu.cn

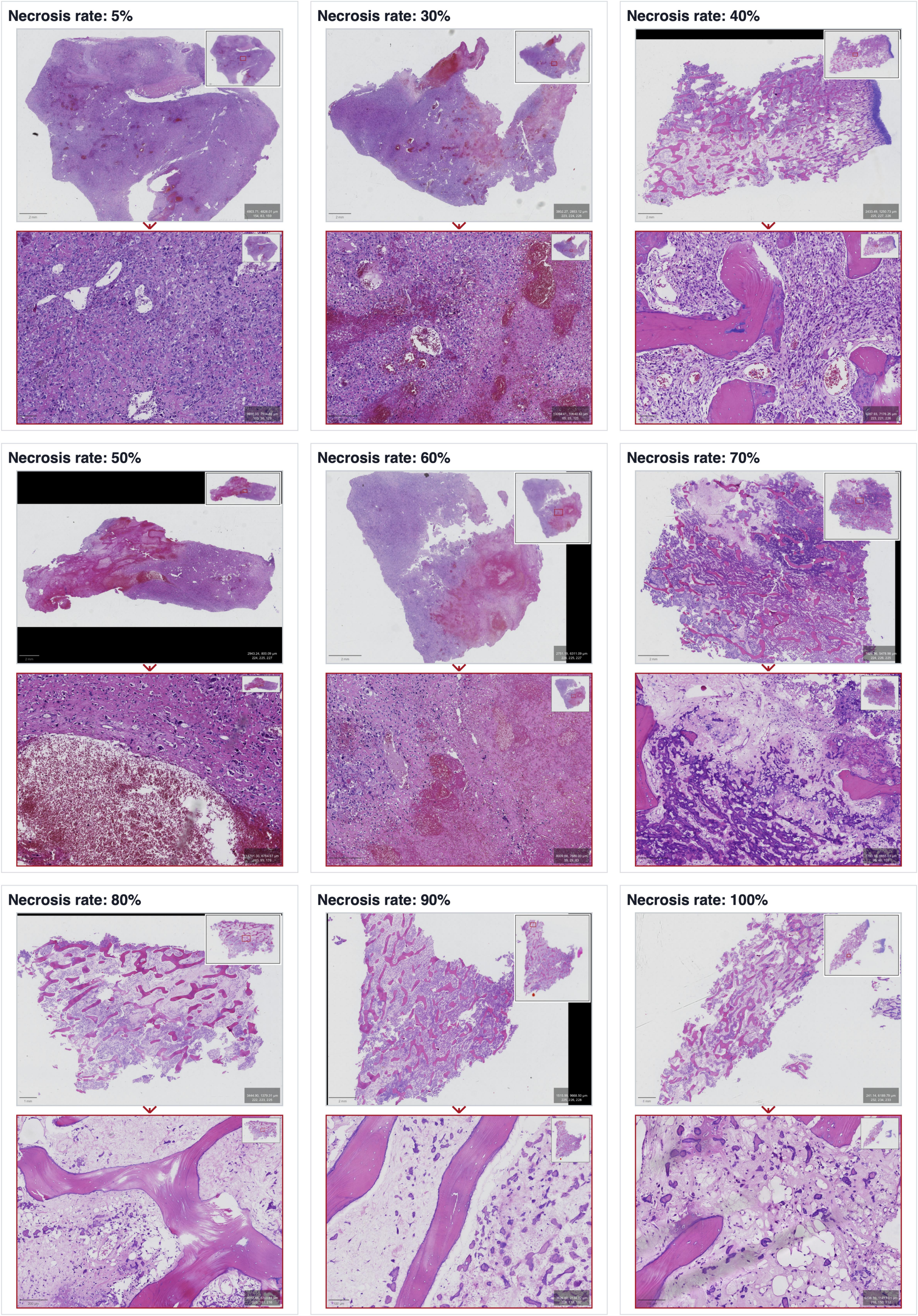

Supplementary Figure 1. Representative H&E-stained sections illustrating different degrees of post-chemotherapy tumor necrosis in osteosarcoma. Representative overview and matched higher-magnification histologic images from osteosarcoma resection specimens showing a spectrum of tumor necrosis rates after neoadjuvant chemotherapy. In each panel, the inset on the overview image indicates the region shown at higher magnification below, highlighting the spatial correspondence between the low-magnification and enlarged views.

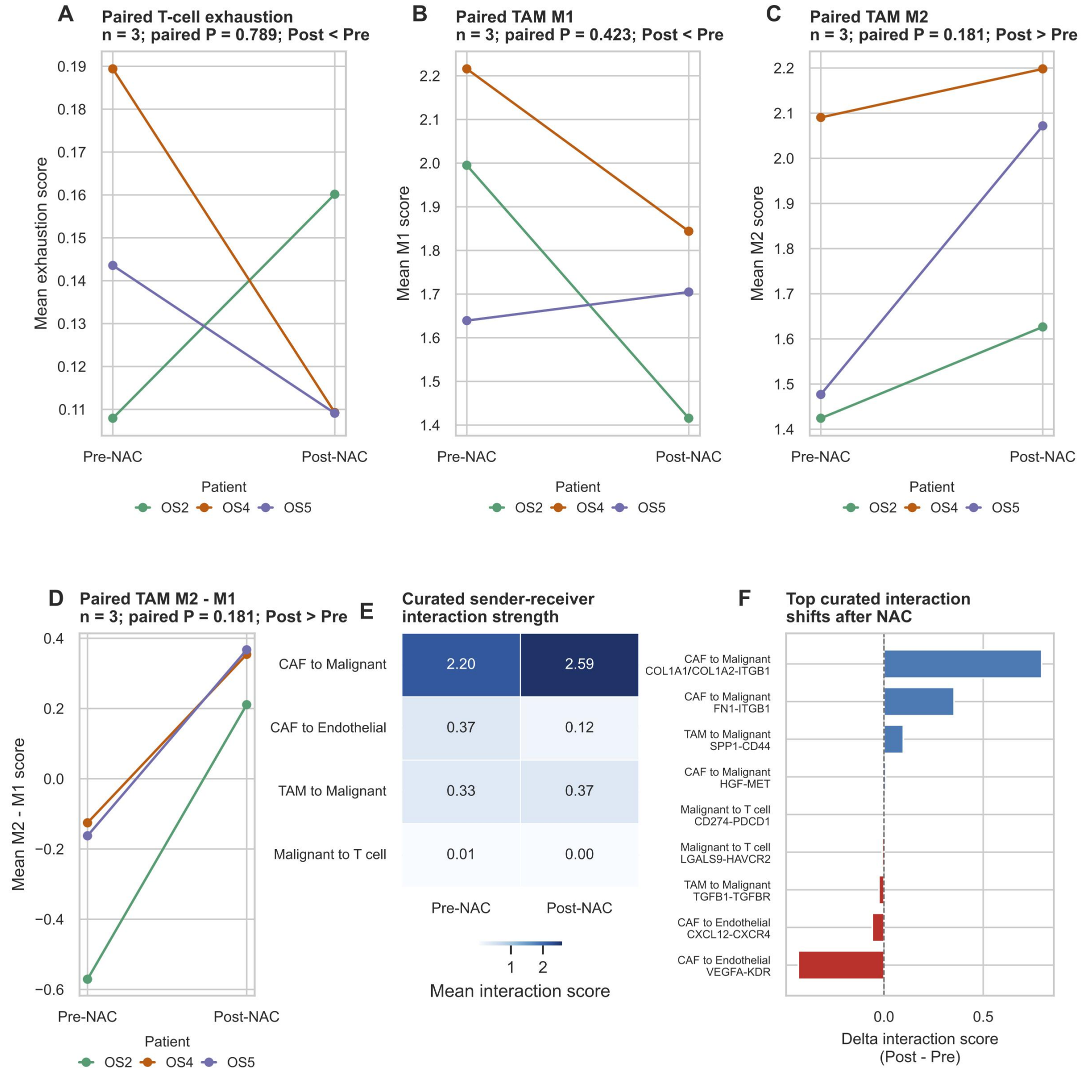

Supplementary Figure 2. Paired tumor microenvironment remodeling after neoadjuvant chemotherapy. (A) Paired T-cell exhaustion score in pre-NAC and post-NAC samples; (B) paired TAM M1 score in pre-NAC and post-NAC samples; (C) paired TAM M2 score in pre-NAC and post-NAC samples; (D) paired TAM M2 minus M1 score in pre-NAC and post-NAC samples, where positive values indicate relative enrichment of the M2-like macrophage program over the M1-like program; (E) heatmap of curated sender-receiver interaction strength across major paired tumor-microenvironment axes; (F) ranked changes in curated ligand-receptor interactions after NAC. Paired statistical comparisons in panels A-D were assessed using two-sided paired Wilcoxon signed-rank tests. CAF subtype scores were not included because pre-NAC CAF abundance was too limited for robust paired subtype inference.

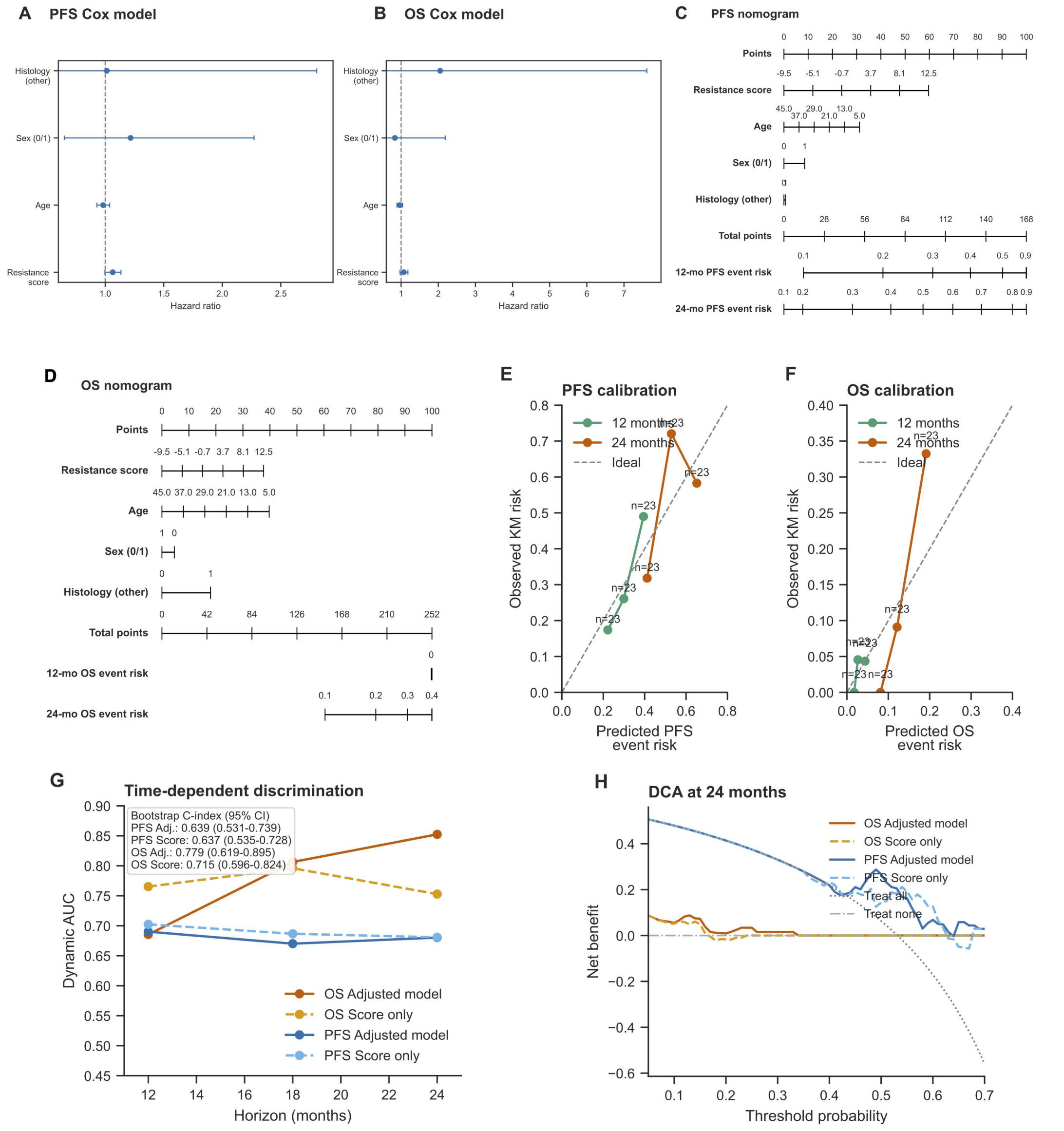

Supplementary Figure 3. PKPH multivariable prognostic modeling, nomograms, calibration, and decision-curve analysis. (A) Forest plot of the multivariable progression-free survival (PFS) model. Covariates included resistance score, age, sex, and histologic subtype grouped as classic versus other. Hazard ratios and 95% confidence intervals were estimated using Cox proportional hazards regression; (B) forest plot of the multivariable overall survival (OS) model using the same observed covariates as in panel A. Hazard ratios and 95% confidence intervals were estimated using Cox proportional hazards regression; (C) nomogram derived from the PKPH PFS multivariable model; (D) nomogram derived from the PKPH OS multivariable model; (E) calibration plot for the PKPH PFS multivariable model at 12 and 24 months. Points summarize tertile-based mean predicted risk versus Kaplan-Meier observed risk, and the dashed diagonal indicates ideal calibration; (F) calibration plot for the PKPH OS multivariable model at 12 and 24 months, displayed using the same tertile-based approach; (G) time-dependent discrimination curves comparing the multivariable model and the score-only model across 12, 18, and 24 months for both PFS and OS, with bootstrap C-index summaries (1,000 resamples); (H) decision-curve analysis at 24 months for the multivariable model and the score-only model for both endpoints. This figure is restricted to observed PKPH covariates only.

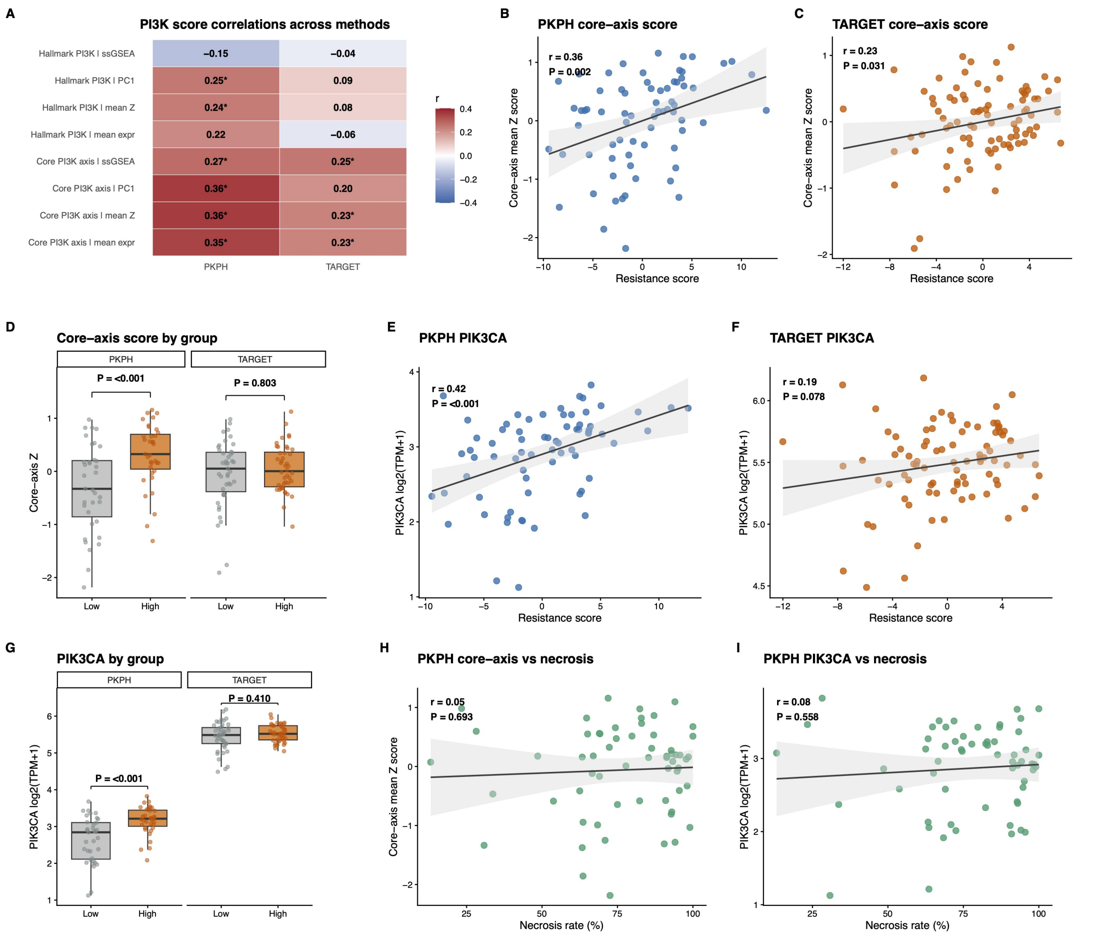

Supplementary Figure 4. Multimethod PI3K scoring, median-split comparison, and necrosis-associated analysis. (A) Heatmap summarizing Pearson correlations between the resistance score and PI3K-related scores across cohorts and scoring methods; $^{*}P < 0.05$; $^{**}P < 0.01$; $^{***}P < 0.001$; $^{****}P < 0.0001$; (B) correlation between the resistance score and the core PI3K-axis mean Z score in the PKPH cohort; (C) correlation between the resistance score and the core PI3K-axis mean Z score in the TARGET cohort; (D) boxplot comparison of the core PI3K-axis mean Z score between resistance-score-high and resistance-score-low groups in PKPH and TARGET; (E) correlation between the resistance score and PIK3CA expression in the PKPH cohort; (F) correlation between the resistance score and PIK3CA expression in the TARGET cohort; (G) boxplot comparison of PIK3CA expression between resistance-score-high and resistance-score-low groups in PKPH and TARGET; (H) correlation between the histologic necrosis rate and the core PI3K-axis mean Z score in the PKPH cohort; (I) correlation between the histologic necrosis rate and PIK3CA expression in the PKPH cohort. Broad pathway scores were calculated using mean Z score, mean expression, principal component 1, and ssGSEA. The compact core PI3K-axis gene set included PIK3CA, PIK3CB, AKT1, AKT2, MTOR, PDPK1, RPTOR, and RICTOR. For panels A, B, C, E, F, H, and I, correlations were assessed by two-sided Pearson correlation tests; for panels D and G, group comparisons used two-sided Wilcoxon rank-sum tests.

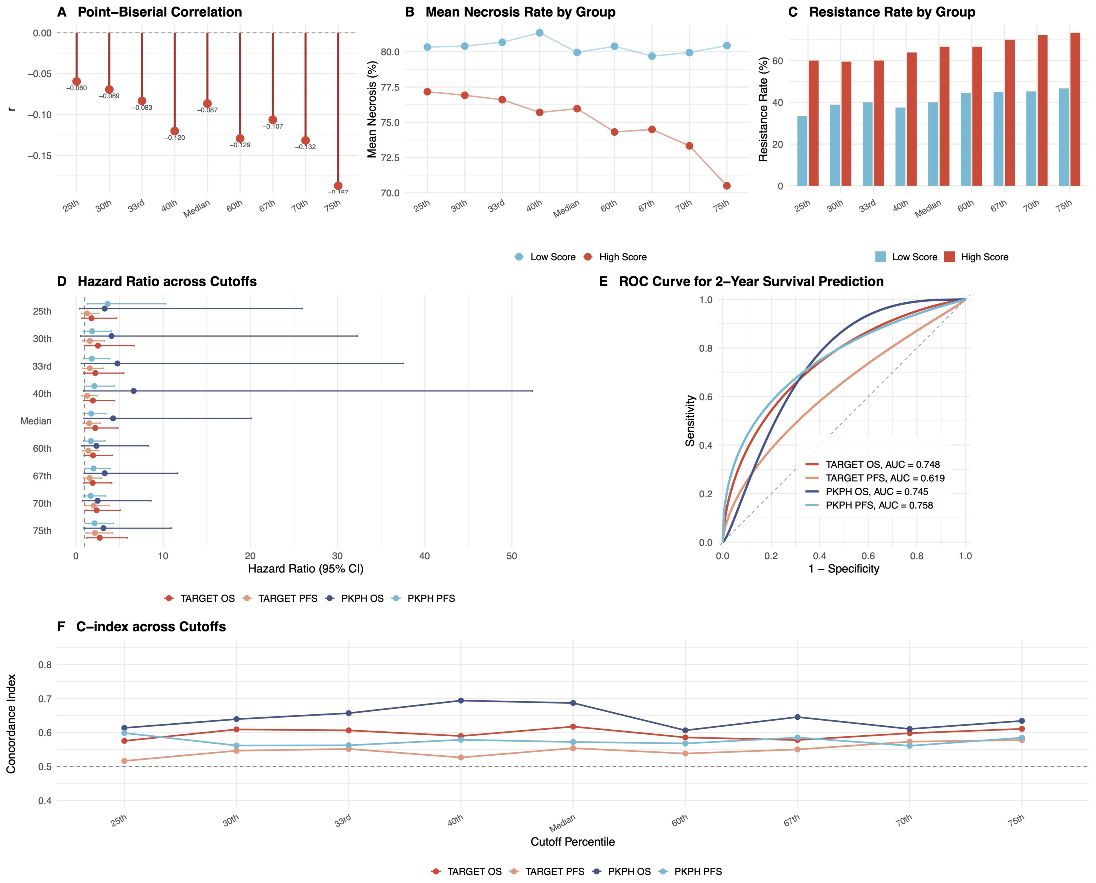

Supplementary Figure 5. Robustness of the nine-gene resistance score across different cutoff settings. (A) Point-biserial correlation between score-defined groups (high vs. low) and tumor necrosis rate across different cutoff percentiles in the PKPH cohort; (B) Mean tumor necrosis rates in the high- and low-score groups across different cutoff percentiles in the PKPH cohort; (C) Proportion of poor responders (tumor necrosis < 90%) in the high- and low-score groups across different cutoff percentiles in the PKPH cohort; (D) Forest plot of hazard ratios for overall survival (OS) and progression-free survival (PFS) across different cutoff settings in the PKPH and TARGET cohorts. Hazard ratios were estimated using univariable Cox proportional hazards regression, and P values were derived from two-sided log-rank tests; (E) Receiver operating characteristic (ROC) curves of the nine-gene score for 2-year survival prediction in different cohorts and endpoints; (F) Concordance index (C-index) of the nine-gene score for OS and PFS across different cutoff settings in the PKPH and TARGET cohorts.
